# First description of the female and morphological variations with range extension of *Kurixaluslenquanensis* (Anura, Rhacophoridae)

**DOI:** 10.3897/BDJ.12.e130411

**Published:** 2024-08-21

**Authors:** Chunyi Pang, Shangjing Tang, Guohua Yu, Jia-Jun Zhou

**Affiliations:** 1 Key Laboratory of Ecology of Rare and Endangered Species and Environmental Protection (Guangxi Normal University), Ministry of Education, Guilin, China Key Laboratory of Ecology of Rare and Endangered Species and Environmental Protection (Guangxi Normal University), Ministry of Education Guilin China; 2 Guangxi Key Laboratory of Rare and Endangered Animal Ecology, College of Life Science, Guangxi Normal University, Guilin, China Guangxi Key Laboratory of Rare and Endangered Animal Ecology, College of Life Science, Guangxi Normal University Guilin China; 3 Zhejiang Forest Resource Monitoring Center, Hangzhou, China Zhejiang Forest Resource Monitoring Center Hangzhou China; 4 Zhejiang Forestry Survey Planning and Design Company Limited, Hangzhou, China Zhejiang Forestry Survey Planning and Design Company Limited Hangzhou China

**Keywords:** *Kurixalus*, 16S rRNA, Yunnan, phylogeny

## Abstract

**Background:**

*Kurixaluslenquanensis* Yu, Wang, Hou, Rao and Yang, 2017 was originally described, based on 14 adult male specimens from Lengquan Village, Mengzi, Yunnan, China. So far, this species is known only from south-eastern Yunnan and information on females of this species is not available. During the field surveys in 2023, two *Kurixalus* specimens (one female and one male) were collected from central eastern Yunnan (Shilin, Kunming, Yunnan, China). These two specimens were confirmed to be *K.lenquanensis* by molecular phylogenetic analyses, based on 16S rRNA sequences.

**New information:**

A female specimen of *K.lenquanensis* is described for the first time and morphological variation amongst populations of this species is provided. The diagnosis and distribution of this species are updated.

## Introduction

The genus *Kurixalus* Ye, Fei and Dubois in Fei, 1999 is widely distributed in eastern India, Indochina, southern China, Malaysia and Philippines (Frost 2024). The taxonomy within *Kurixalus* was once very confusing owing to morphological conservativeness (Yu et al. 2017). In recent years, with the in-depth field surveys and use of integrative taxonomic methods, the underestimated species diversity of *Kurixalus* has been gradually revealed (e.g. Yu et al. (2017), Yu et al. (2018), Nguyen et al. (2020), Zeng et al. (2021), Hou et al. (2021)). At the present, the genus *Kurixalus* contains 24 species (Frost 2024), 13 of which are recorded in China (Xizang, Yunnan, Sichuan, Guizhou, Guangxi, Guangdong, Hainan, Zhejiang and Taiwan), namely *K.berylliniris* Wu, Huang, Tsai, Li, Jhang & Wu, 2016, *K.bisacculus* (Taylor, 1962), *K.eiffingeri* (Boettger, 1895), *K.hainanus* (Zhao, Wang & Shi, 2005), *K.idiootocus* (Kuramoto & Wang, 1987), *K.inexpectatus* Messenger, Yang, Borzée, Chuang & Othman, 2022, *K.lenquanensis* Yu, Wang, Hou, Rao & Yang, 2017, *K.naso* (Annandale, 1912), *K.odontotarsus* (Ye & Fei, 1993), *K.raoi* Zeng, Wang, Yu & Du, 2021, *K.silvaenaias* Hou, Peng, Miao, Liu, Li & Orlov, 2021, *K.wangi* Wu, Huang, Tsai, Li, Jhang & Wu, 2016 and *K.yangi* Yu, Hui, Rao & Yang, 2018 (AmphibiaChina 2024). However, recently, Lyu et al. (2024) considered that *K.qionglaiensis* Guo, Zhong, Leung, Wang & Hu, 2022 has priority over *K.silvaenaias* and *K.inexpectatus* is a junior synonym of *K.idiootocus*.

*Kurixaluslenquanensis* is endemic to Yunnan, China and is sister to the clade of *K.raoi*, *K.silvaenaias*, *K.idiootocus* and *K.inexpectatus* (Mo et al. 2023). It was described by Yu et al. (2017), based on 14 male specimens from Honghe Hani and Yi Autonomous Prefecture, Yunnan, China, and currently is known only from south-eastern Yunnan (Mengzi, Gejiu and Wenshan; AmphibiaChina (2024)). According to the original description (Yu et al. 2017), *K.lenquanensis* is characterised by small body size (mean SVL 27 mm in males), obtusely pointed snout, chin clouded with brown, dorsal surface brownish mixed with dark marking, iris golden, nuptial pad slight, vomerine teeth present and single internal vocal sac. So far, female information about this species is unavailable and morphological variation amongst populations of this species has never been reported.

**Figure 1.**
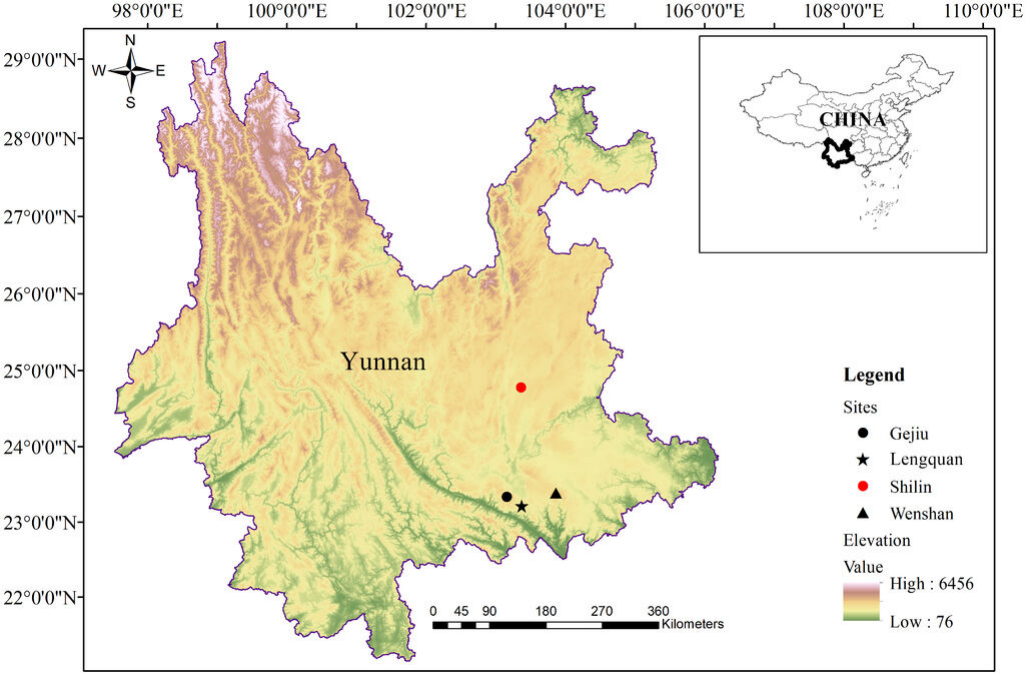
Distribution of *Kurixaluslenquanensis* in Yunnan, China. The star indicates the type locality and the red circle represents the newly-discovered population in Shilin.

During a herpetofaunal survey of central eastern Yunnan in 2023, two adult specimens (one female and one male) of *Kurixalus* were collected from Shilin, Kunming (Fig. 1). Molecular phylogenetic analyses, based on the 16S rRNA gene, confirmed that these two specimens belong to *K.lenquanensis*, but, morphologically, these two specimens show some differences from other populations of this species. Here, we provide first morphological description of the female *K.lenquanensis*, investigate morphological variation amongst populations and extend the distribution of this species from south-eastern Yunnan to central eastern Yunnan, China.

## Materials and methods

Specimens were collected during fieldwork in Shilin, Yunnan, China in 2023. Sex was determined, based on whether the vocal sac opening presents on the floor of the mouth at each corner. Specimens were photographed, euthanised, fixed and then stored in 75% ethanol without formalin fixation. Live tissues were taken and preserved in 99% alcohol. Specimens were deposited at Guangxi Normal University (**GXNU**).

Morphometric data were taken using electronic digital calipers to the nearest 0.1 mm. Morphological terminology followed Yu et al. (2017). A total of five specimens (two from Shilin and three from Wenshan) were measured. Measurements included: snout vent length (SVL, from tip of snout to vent); head length (HL, from tip of snout to rear of jaws); head width (HW, width of head at its widest point); snout length (SL, from tip of snout to anterior corner of eye); internarial distance (IND, distance between nares); interorbital distance (IOD, minimum distance between upper eyelids); upper eyelid width (UEW, maximum width of upper eyelid); eye diameter (ED, diameter of exposed portion of eyeball); distance between nostril and eye (DNE, from nostril to anterior border of eye); tympanum diameter (TD, the greater of tympanum vertical and horizontal diameters); forearm and hand length (FHL, from elbow to tip of third finger); tibia length (TL, distance from knee to heel); foot length (FL, from proximal end of inner metatarsal tubercle to tip of fourth toe); and length of foot and tarsus (TFL, from tibiotarsal joint to tip of fourth toe). Webbing formula followed Myers and Duellman (1982).

Measurements were corrected for size (measurements divided by SVL) and morphometric data of types of *K.lenquanensis* was retrieved from Yu et al. (2017). To investigate morphological variation amongst populations, we conducted a principal component analysis (PCA), based on a correlation matrix of size-corrected measurements using SPSS v.17.0 (SPSS Inc., Chicago, IL, USA). Scatter plot of the first two PCA factors was used to examine the morphological differentiation between specimens from different populations.

The classifications of AmphibiaChina (2024) and Frost (2024) were followed. Total genomic DNA was extracted from liver tissues stored in 99% ethanol. A fragment encoding mitochon­drial 16S rRNA gene was ampli­fied and sequenced using the primers L2188 (Matsui et al. 2006)/16H1 (Hedges 1994). The experiment protocols are the same as those described in Yu et al. (2010). Two samples from Shilin, Yunnan, four samples from Wenshan, Yunnan and an individual of *K.inexpectatus* from Zhejiang were newly sequenced and all new sequences have been deposited in GenBank under Accession Nos. PQ056924‒PQ056930 (Table 1).

Phylogenetic relationships within *Kurixalus* were inferred from the 16S rRNA gene. Homologous sequences of known *Kurixalus* species and outgroups were obtained from GenBank (Table 1). *Raorchestesmenglaensis* and *Philautusabditus* were used as outgroups according to Yu et al. (2017). Sequences were aligned using MUSCLE with default parameters in MEGA v.7.0 (Kumar et al. 2016), then checked by eye for accuracy. The best substitution model was selected using the correct­ed Akaike Information Criterion (AICc) in jModelTest v.2.1.10 (Darriba et al. 2012). Phylogenetic analyses were conducted using two methods. Bayesian Inference (BI) was performed in MrBayes v.3.2.6 (Ronquist et al. 2012) un­der the selected substitution model (GTR + I + G). Two runs were performed simultaneously with four Markov chains starting from a random tree. The chains were run for 3,000,000 generations and sampled every 100 gener­ations. The first 25% of the sampled tree was discarded as burn-in after the standard deviation of split frequencies of the two runs was less than 0.01. The remaining trees were then used to create a consensus tree and to estimate Bayesian posterior probabilities (BPPs). In addition, Maximum Likelihood (ML) analysis was conducted in raxmlGUI v.2.0 (Edler et al. 2021) with 1000 rapid bootstrap replicates.

## Taxon treatments

### Kurixalus lenquanensis

Yu, Wang, Hou, Rao & Yang, 2017

4F283E26-59B0-51DE-B24A-7CD71F9B8C7B

#### Materials

- **Type status:** Other material. **Occurrence:** catalogNumber: GXNU YU000597; individualCount: 1; sex: female; occurrenceID: 99FBB6BB-F3A0-5070-B11A-51F13A335C63; **Taxon:** scientificName: *Kurixaluslenquanensis*; **Location:** country: China; stateProvince: Yunnan; county: Shilin; verbatimElevation: 1876 m; verbatimCoordinates: 24°46'39''N, 103°21'51''E
- **Type status:** Other material. **Occurrence:** catalogNumber: GXNU YU000598; individualCount: 1; sex: male; occurrenceID: B60EC082-E961-542F-999A-A70E88161376; **Taxon:** scientificName: *Kurixaluslenquanensis*; **Location:** country: China; stateProvince: Yunnan; county: Shilin; verbatimElevation: 1876 m; verbatimCoordinates: 24°46'39''N, 103°21'51''E
- **Type status:** Other material. **Occurrence:** catalogNumber: GXNU YU20170001; individualCount: 1; sex: male; occurrenceID: 9FDD5697-583E-5B7E-AC36-202FAC93303B; **Taxon:** scientificName: *Kurixaluslenquanensis*; **Location:** country: China; stateProvince: Yunnan; county: Wenshan; verbatimElevation: 1760 m; verbatimCoordinates: 23°23'2''N, 103°52'1''E
- **Type status:** Other material. **Occurrence:** catalogNumber: GXNU YU20170002; individualCount: 1; sex: male; occurrenceID: B1ACC73F-FCE6-54D2-AC04-AFB5E55971B9; **Taxon:** scientificName: *Kurixaluslenquanensis*; **Location:** country: China; stateProvince: Yunnan; county: Wenshan; verbatimElevation: 1760 m; verbatimCoordinates: 23°23'2''N, 103°52'1''E
- **Type status:** Other material. **Occurrence:** catalogNumber: GXNU YU20170003; individualCount: 1; sex: male; occurrenceID: 2FB11595-CD29-51D5-88F5-5E712B937681; **Taxon:** scientificName: *Kurixaluslenquanensis*; **Location:** country: China; stateProvince: Yunnan; county: Wenshan; verbatimElevation: 1760 m; verbatimCoordinates: 23°23'2''N, 103°52'1''E

#### Description of the female specimen (GXNU YU000597)

**Figure 2.**
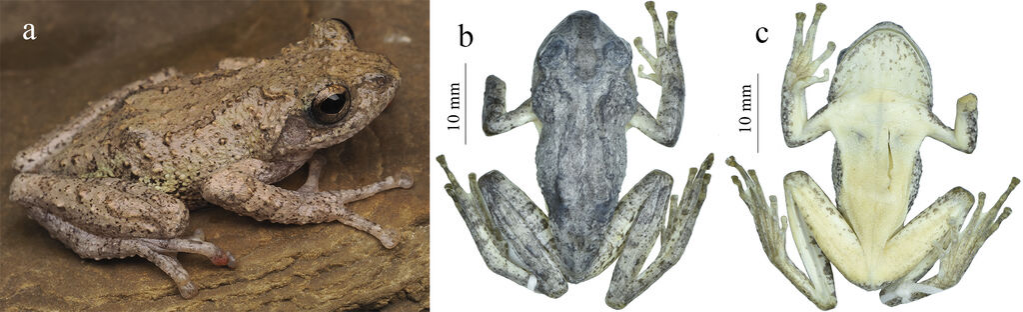
*Kurixaluslenquanensis* (GXNU YU000597), female from Shilin, Yunnan, China; **a** life, lateral view; **b, c** in preservative, dorsal (b) and ventral (c) views.

Adult female (Fig. 2), SVL 34.2 mm (Table 2), head length (HL) shorter than head width (HW), HL 89.3% of HW; snout obtusely pointed, no dermal prominence on tip, projecting slightly beyond margin of lower jaw in ventral view; SL (4.4 mm) slightly longer than ED (4.1 mm); canthus rostralis blunt and curved; lore region oblique, slightly concave; nostril oval, slightly protuberant, closer to tip of snout than to eye; IND (3.1 mm) slightly narrower than IOD (3.8 mm) and UEW (3.5 mm); pineal spot absent; pupil oval, horizontal; tympanum dis­tinct (TD 2.0 mm), rounded, nearly equal to half ED; supratympanic fold distinct, curves from posterior edge of eye to insertion of arm; vomerine teeth in two oblique patches touching inner front edges of oval choanae; tongue notched posteriorly.

Limbs slender; relative length of fingers I < II < IV < III; tips of all four fingers expanded into discs with circum-marginal and transverse ventral grooves; fingers webbed at base; subarticular tubercles rounded, formula 1, 1, 2, 2, the distal one more prominent than the proximal one on fingers III and IV; two metacarpal tubercles, the outer one divided into two; a row of white warts forming serrated fringe along outer edge of forearm.

Heels overlapping when legs at right angle to body; relative length of toes I < II < V = III < IV; tips of toes expanded into discs with circum-marginal and transverse ventral grooves; toes webbed, webbing formula I2‒2II1.5–3III2‒3IV3‒2V; subarticular tubercles prominent and rounded, formula 1, 1, 2, 3, 2; a series of white tubercles forming serrated dermal fringe along outer edge of tarsus and fifth toe; inner metatarsal tubercle oval, outer metatarsal tubercle absent.

Numerous tubercles scattered on top of head, upper eyelids, dorsum, flanks and dorsal surface of limbs; a few white tubercles below vent; throat and chest finely granular and abdomen coarsely granular; dorsal surface of limbs tuberculate and ventral surface of thighs finely granular.

Iris golden, mottled with black washing; dorsal surface greyish-brown with dark brown saddle-shaped mark on dorsum, beginning behind eye; dark brown invert­ed triangular-shaped mark between eyes; lateral head and tympanic region brown with dark brown spot below canthus; broad dark brown bar along canthus rostralis; flanks brown, mottled with greyish-green; limbs dorsally brown with clear dark brown barring; rear, anterior and venter of thigh light yellow with scattered brown spots; many dark spots on dorsum, flanks and dorsal surface of limbs; chest and abdomen white, immaculate; chin scattered with dark blotches.

#### Extended diagnosis of *K.lenquanensis*

Body size small (adult males 22.1‒28.9 mm, female 34.2 mm); tips of fingers and toes enlarged to discs, bearing circum-marginal grooves; finger webbing poorly developed and toe webbing moderately developed; ser­rated dermal fringes along outer edge of forearm and tarsus; an inverted triangular-shaped dark brown mark between eyes; “) (” saddle-shaped or X-shaped dark brown marking on dorsum; dorsal and lateral surfaces coarse with small and irregular tubercles; obtusely pointed snout with no prominence on tip; curved canthus rostralis; males have slight nuptial pad on the base of first finger; dorsal colour greyish-brown; chin clouded with brown or scattered with brown patches or nearly immaculate; ventral surface nearly immaculate or scattered with brown patches; vomerine teeth present; iris gold brown with black washing; males have single internal vocal sac; dermal fringes along outer edge of limbs; rough flanks; and fine granular throat and chest.

#### Extended distribution

**Figure 3.**
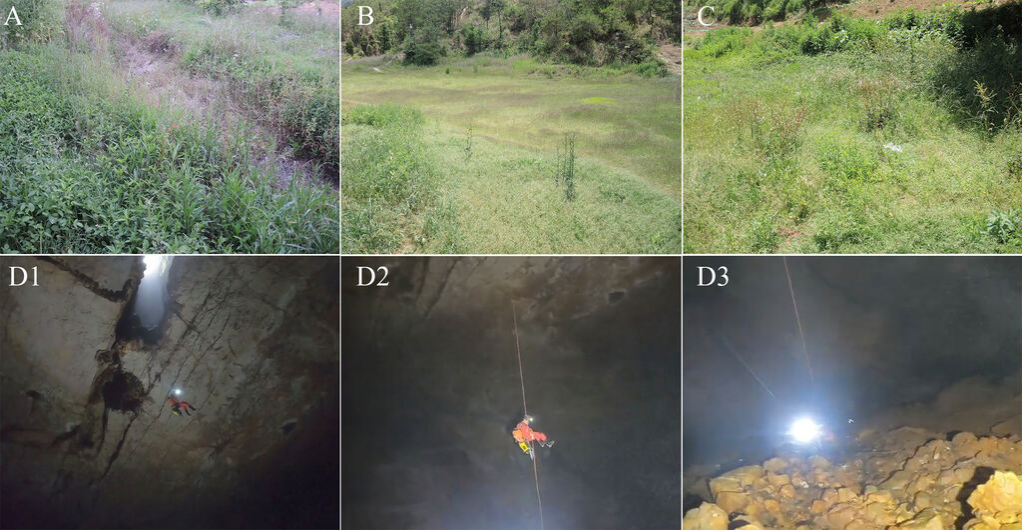
Habitats of *K.lenquanensis* in Mengzi (A), Gejiu (B), Wenshan (C) and Shilin(D).

*Kurixaluslenquanensis* is currently known from south-eastern Yunnan (Mengzi, Gejiu, Wenshan) and central eastern Yunnan (Shilin), China. This species was found on shrubs in a fruit garden at Lengquan Villiage in Mengzi, shrubs near Yangjiatian Reservoir in Gejiu and shrubs at Yigebai Villiage in Wenshan (Fig. 3). However, differing from the habitats of this species in south-eastern Yunnan, in Shilin, the species was found in a sink hole (Fig. 3) away from farmland during the extremely dry season in 2023. The cave is about 100 m in vertical depth and the environment inside it is moist. Vegetation was rich at the entrance of the cave, but no vegetation and water were inside the cave. Probably the cave acts as a refuge of *K.lenquanensis* during the dry season because of the moist environment inside it.

## Analysis

**Figure 4.**
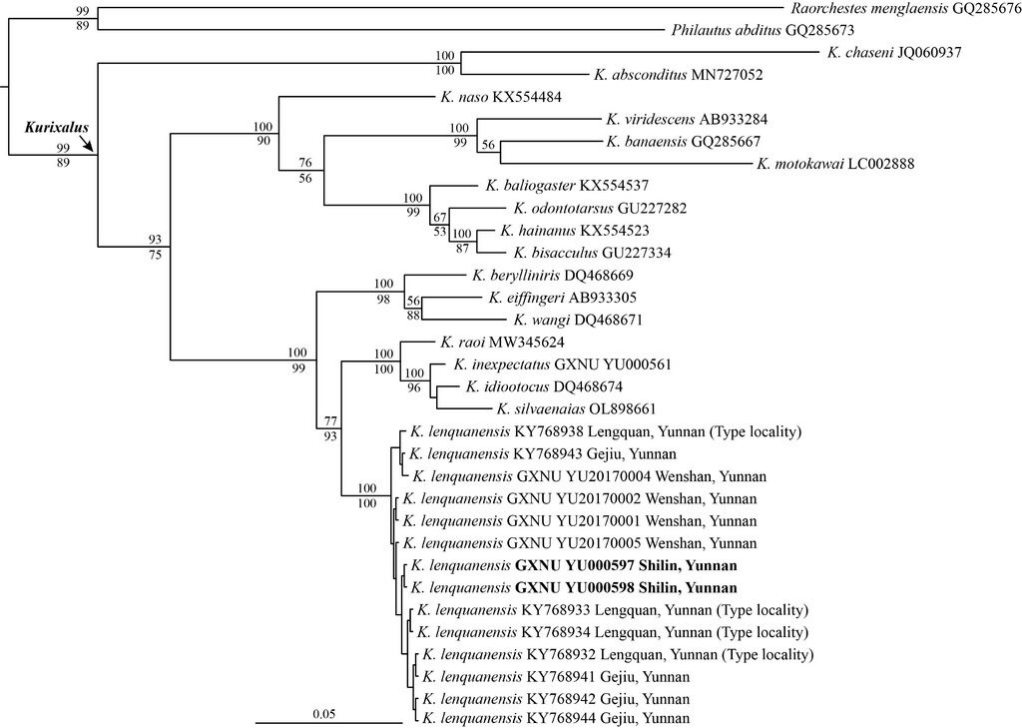
Bayesian phylogeny of *Kurixalus* inferred from 16S rRNA sequences. The numbers above and below the branches are Bayesian posterior probabilities (BPP) and Maximum Likelihood (ML) bootstrap values, respectively (only values greater than 50% are shown).

Bayesian Inference and ML analysis obtained consistent topology. The two newly-collected specimens from Shilin, Yunnan clustered together with specimens of *K.lenquanensis* (including types of this species) with strong support values (Fig. 4). The genetic distance (uncorrected p-distance) between specimens from Shilin and *K.lenquanensis* from other sites in 16S sequences was only 0.2% (Table 3).

**Figure 5.**
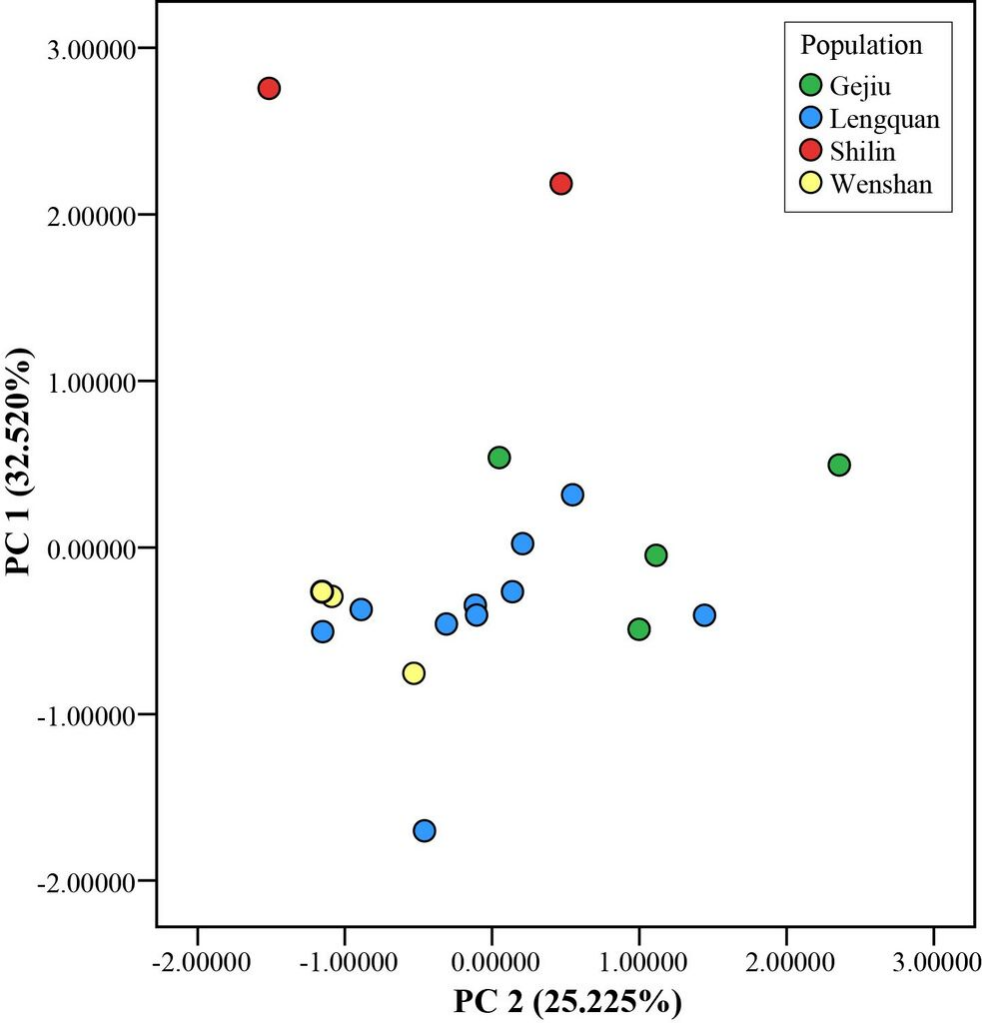
Scatter plot of the principal component analysis, based on size-adjusted morphological data of *Kurixaluslenquanensis* from different populations.

**Figure 6.**
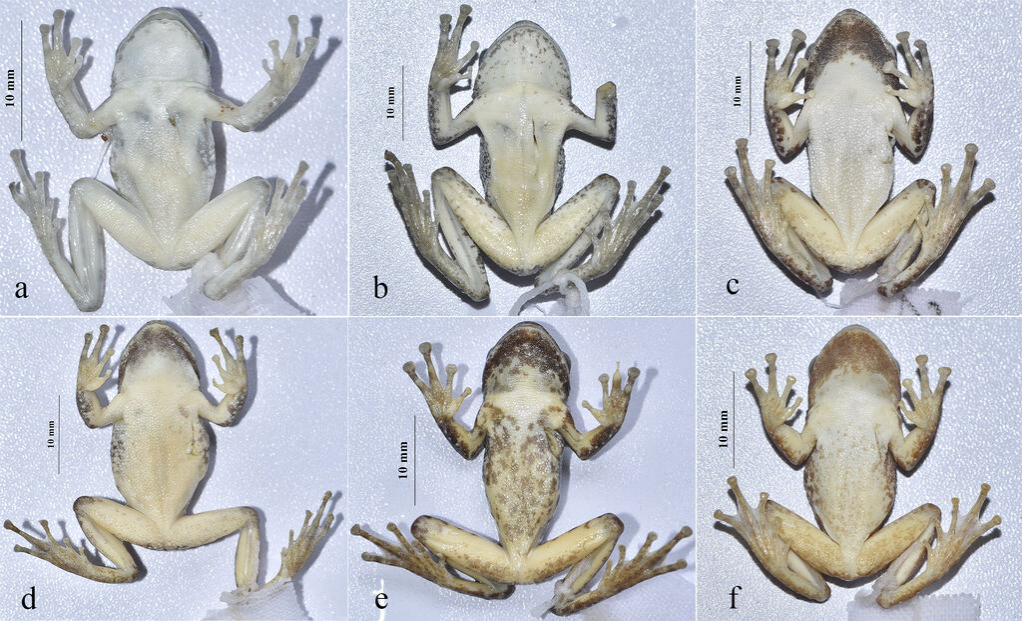
*Kurixaluslenquanensis*, ventral views from Shilin (a, GXNU YU000598; b, GXNU YU000597), Lengquan (c, GXNU YU20160046), Wenshan (d, GXNU YU20170001; e, GXNU YU20170003) and Gejiu (f, GXNU YU20160137), Yunnan, China.

Morphological data of specimens from Shilin and *K.lenquanensis* from Wenshan are summarised in Table 2. PCA analysis revealed that the first two principal components accounted for 57.745% of the total variance (Table 4) and loadings for PC1 were heavily loaded on FHL and TL, which separated Shilin specimens from other specimens of *K.lenquanensis* (Fig. 5). Additionally, the chin of the two specimens from Shilin is not clouded with dark, which is different from specimens collected from Lengquan (type locality), Gejiu and Wenshan (Fig. 6). The chin of the female specimen (GXNU YU000597) is scattered with a few dark patches, whereas the chin of the male specimen (GXNU YU000598) is nearly immaculate.

## Discussion

Our molecular phylogenetic analyses, based on 16S rRNA sequences, confirmed that the two specimens from Shilin, Yunnan belong to *K.lenquanensis*. Although the genetic distance between specimens from Shilin and specimens from other population in 16S rRNA is very low (0.2%) and, morphologically, the two specimens from Shilin are broadly in line with the original description of *K.lenquanensis* (Yu et al. 2017), the two individuals from Shilin population show some morphological differences from other populations. Firstly, compared to other populations, the Shilin population has longer tibia, forearm and hand (Fig. 5). Secondly, differing from specimens from Lengquan, Gejiu and Wenshan that are clouded with brown on the skin of the chin, the two specimens from Shilin have no clouded brown on the skin of the chin; the chin of the male specimen GXNU YU000598 is nearly immaculate and the chin of the female specimen GXNU YU000597 is scattered with a few brown spots (Fig. 6). Considering that only two samples from Shilin were examined in this study, further studies, based on more specimens from Shilin, are needed to investigate whether these morphological variations are stable or not.

In addition, we observed that the colour pattern of the venter of *K.lenquanensis* also varies amongst specimens. It was known that *K.lenquanensis* has no large dark patches on the ventral surface (Yu et al. 2017). However, the specimen GXNU YU20170003 collected from Wenshan has distinct dark patches on the venter (Fig. 6). Based on this information of morphological variations, we have provided an extended diagnosis of this species which will be helpful for the identification of this species in the field.

*Kurixaluslenquanensis* was previously known, based on only adult males from south-eastern Yunnan. This study reports the female specimen of this species for the first time and extends the distribution of *K.lenquanensis* from south-eastern Yunnan northwards to central eastern Yunnan. Considering the obvious geographical gap in distribution between the newly-discovered population in central eastern Yunnan (Shilin) and the three known populations in south-eastern Yunnan (Lengquan, Gejiu and Wenshan; Fig. 1), it could be expected that more populations of *K.lenquanensis* would be discovered in the intermediate region.

## Supplementary Material

XML Treatment for Kurixalus lenquanensis

## Figures and Tables

**Table 1.** Samples used in phylogenetic analyses in this study.

Species	Voucher	Locality (ID)	Accession No.
*Raorchestesmenglaensis*	060821286Rao	Lvchuan, Yunnan, China	GQ285676
*Philautusabditus*	ROM 33145	Krong Pa, Gia Lai, Vietnam	GQ285673
*K.chaseni*	FMNH267896	Sarawak, Malaysia	JQ060937
*K.absconditus*	MZB21862	Borneo, Indonesia	MN727052
*K.naso*	Rao06301	Xizang, China	KX554484
*K.viridescens*	VNMN 03802	Hon Ba, Khanh Hoa, Vietnam	AB933284
*K.banaensis*	ROM 32986	Krong Pa, Gia Lai, Vietnam	GQ285667
*K.motokawai*	VNMN 03458	Kon Tum, Vietnam	LC002888
*K.baliogaster*	ROM29860	Gia Lai, Vietnam	KX554537
*K.odontotarsus*	YGH 090175	Mengyang, Yunnan, China	GU227282
*K.hainanus*	Rao14111301	Hainan, China	KX554523
*K.bisacculus*	THNHM 10051	Pua, Nan, Thailand	GU227334
*K.berylliniris*	11311 (CE01X)	Beinan, Taitung, Taiwan, China	DQ468669
*K.eiffingeri*	KUHE 12910	Okinawa Islands, Japan	AB933305
*K.wangi*	11328 (CE06)	Shouka, Pintung, Taiwan, China	DQ468671
*K.raoi*	GXNUYU140145	Xingyi, Guizhou, China	MW345624
*K.inexpectatus*	GXNU YU000561	Changxing, Zhejiang, China	PQ056924
*K.idiootocus*	A127	Wulai, Taipei, Taiwan, China	DQ468674
*K.silvaenaias*	CIB118054	Qionglai, Sichuan, China	OL898661
*K.lenquanensis*	KIZ 170182Y	Lengquan, Mengzi, Yunnan, China	KY768938
*K.lenquanensis*	KIZ 170185Y	Yangjiatian, Gejiu, Yunnan, China	KY768943
*K.lenquanensis*	GXNU YU20170001	Wenshan, Yunnan, China	PQ056925
*K.lenquanensis*	GXNU YU20170002	Wenshan, Yunnan, China	PQ056926
*K.lenquanensis*	GXNU YU20170004	Wenshan, Yunnan, China	PQ056927
*K.lenquanensis*	GXNU YU20170005	Wenshan, Yunnan, China	PQ056928
*K.lenquanensis*	GXNU YU000597	Shilin, Yunnan, China	PQ056929
*K.lenquanensis*	GXNU YU000598	Shilin, Yunnan, China	PQ056930
*K.lenquanensis*	KIZ 170176Y	Lengquan, Mengzi, Yunnan, China	KY768932
*K.lenquanensis*	KIZ 170177Y	Lengquan, Mengzi, Yunnan, China	KY768933
*K.lenquanensis*	KIZ 170178Y	Lengquan, Mengzi, Yunnan, China	KY768934
*K.lenquanensis*	KIZ 170183Y	Gejiu, Yunnan, China	KY768941
*K.lenquanensis*	KIZ 170184Y	Gejiu, Yunnan, China	KY768942
*K.lenquanensis*	KIZ 170186Y	Gejiu, Yunnan, China	KY768944

**Table 2.** Measurements (mm) of *Kurixaluslenquanensis* from Shilin and Wenshan, Yunnan, China.

Character	GXNU YU000597	GXNU YU000598	GXNU YU20170001	GXNU YU20170002	GXNU YU20170003
Sex	F	M	M	M	M
SVL	34.2	22.1	28.8	28.5	28.7
HL	10.4	7.1	8.6	8.9	8.6
HW	13.1	8.4	10.0	9.9	9.7
SL	4.4	2.9	3.7	3.9	3.7
IND	3.1	2.3	2.8	2.6	2.8
IOD	3.8	2.9	3.1	2.9	3.1
UEW	3.5	2.4	2.8	2.7	2.5
ED	4.1	3.0	3.6	3.7	3.6
TD	2.0	1.2	1.6	1.7	1.4
DNE	2.8	1.9	2.3	2.2	1.9
FHL	19.1	11.4	13.9	13.7	14.1
TL	18.1	11.0	12.2	12.9	13.4
TFL	25.8	15.0	18.9	18.6	19.3
FL	16.7	10.2	13.1	12.1	12.3

**Table 3.** Uncorrected p-distance (%) between *Kurixalus* specimens (GXNU YU000597 and GXNU YU000598) from Shilin, Yunnan, China and *Kurixalus* species, based on 16S rRNA sequences.

Species	1	2	3	4	5	6	7	8	9	10	11	12	13	14	15	16	17	18	19
GXNU YU000597																			
GXNU YU000598	0.0																		
*K.lenquanensis*	0.2	0.2																	
*K.idiootocus*	3.8	3.8	4.0																
*K.raoi*	4.3	4.3	4.4	2.1															
*K.inexpectatus*	4.4	4.4	4.5	0.9	2.4														
*K.silvaenaias*	5.1	5.1	5.2	1.7	3.6	2.3													
*K.berylliniris*	5.3	5.3	5.5	6.3	5.1	5.7	6.1												
*K.wangi*	5.7	5.7	5.9	6.6	5.9	6.1	5.7	4.0											
*K.eiffingeri*	5.9	5.9	6.0	6.1	7.4	7.1	7.5	3.2	3.6										
*K.naso*	10.3	10.4	10.4	9.5	10.7	10.8	11.7	9.5	8.9	11.1									
*K.odontotarsus*	10.9	10.9	11.0	10.4	11.6	11.3	12.2	10.6	10.4	11.4	8.6								
*K.hainanus*	11.2	11.1	11.3	11.0	12.0	11.9	12.6	11.6	11.0	12.2	8.3	3.0							
*K.baliogaster*	11.3	11.2	11.3	11.4	11.5	11.7	12.1	11.8	10.6	11.7	8.4	3.4	2.8						
*K.bisacculus*	11.4	11.4	11.5	11.6	12.1	11.7	12.6	11.8	10.8	11.8	8.9	3.2	1.4	3.3					
*K.banaensis*	12.4	12.4	12.4	11.4	12.4	11.8	13.1	10.8	11.0	11.9	10.8	9.2	9.9	9.9	9.8				
*K.viridescens*	12.9	12.9	12.9	12.5	12.6	12.2	13.1	11.8	11.4	11.7	11.0	9.4	9.8	9.5	9.4	6.1			
*K.motokawai*	14.4	14.4	14.4	12.7	14.1	14.1	15.0	12.7	12.9	13.8	13.0	11.9	12.0	12.1	12.2	8.8	9.4		
*K.absconditus*	15.2	15.2	15.2	14.2	15.2	15.0	15.9	13.3	12.7	15.5	15.1	16.4	16.1	16.2	16.1	14.9	16.2	16.1	
*K.chaseni*	17.2	17.2	17.1	16.8	17.8	18.0	18.1	16.3	17.0	17.0	17.1	17.8	18.0	18.2	18.0	17.0	19.3	18.8	11.5

**Table 4.** Factor loadings of first two principal components of 11 size-adjusted morphometric characteristics of *Kurixaluslenquanensis* from different sites.

Character	PC1	PC2
Eigenvalue	3.577	2.775
% variation	32.520%	25.225%
HL	0.122	0.824
HW	0.756	0.554
SL	‒0.252	0.745
IND	0.146	0.685
IOD	0.717	0.500
UEW	0.539	‒0.256
ED	‒0.362	0.556
TD	‒0.134	0.142
FHL	0.859	‒0.243
TL	0.838	‒0.058
FL	0.716	‒0.243
